# Mopeia Virus–related Arenavirus in Natal Multimammate Mice*,* Morogoro, Tanzania

**DOI:** 10.3201/eid1512.090864

**Published:** 2009-12

**Authors:** Stephan Günther, Guy Hoofd, Remi Charrel, Christina Röser, Beate Becker-Ziaja, Graham Lloyd, Christopher Sabuni, Ron Verhagen, Guido van der Groen, Jan Kennis, Abdul Katakweba, Robert Machang'u, Rhodes Makundi, Herwig Leirs

**Affiliations:** Bernhard-Nocht-Institute for Tropical Medicine, Hamburg, Germany (S. Günther, B. Becker-Ziaja); Institute of Tropical Medicine Leopold II, Antwerp, Belgium (G. Hoofd, G. van der Groen); Université de la Méditerranée, Marseille, France (R. Charrel); Artus Company, Hamburg (C. Röser); Centre for Emergency Preparedness and Response, Salisbury, UK (G. Lloyd); Sokoine University of Agriculture, Morogoro, Tanzania (C. Sabuni, A. Katakweba, R. Machang’u, R. Makundi); University of Antwerp Department of Biology, Antwerp (R. Verhagen, J. Kennis, H. Leirs); University of Aarhus Department of Integrated Pest Management, Kongens Lyngby, Denmark (H. Leirs)

**Keywords:** arenavirus, Mopeia, Tanzania, rodents, Mastomys natalensis, vector-borne infections, viruses, dispatch

## Abstract

A serosurvey involving 2,520 small mammals from Tanzania identified a hot spot of arenavirus circulation in Morogoro. Molecular screening detected a new arenavirus in Natal multimammate mice (*Mastomys natalensis)*, Morogoro virus, related to Mopeia virus. Only a small percentage of mice carry Morogoro virus, although a large proportion shows specific antibodies.

Arenaviruses are segmented negative-strand RNA viruses. Their natural hosts are various rodent species. The virus family comprises several human pathogens causing hemorrhagic fever, namely Machupo, Guanarito, Junin, Sabia, and Chapare viruses in South America, and Lassa and Lujo viruses in Africa (*1*–*3*). In addition, Africa harbors arenaviruses that are not linked with human disease: Mobala, Ippy, Mopeia, and Kodoko viruses (*4*–*7*). We conducted a systematic search in wildlife in Tanzania to identify new African arenaviruses.

## The Study

During 1985 through 1989, a total of 2,520 small mammals were live-trapped in different regions of Tanzania. After species determination, they were measured and bled by orbital puncture. Serum samples were tested by indirect immunofluorescent antibody (IFA) assay (*8*). Lassa virus was used as antigen due to its cross-reactivity with immune sera from animals infected with other arenaviruses (*4*,*6*). Clusters of seropositivity were found in *Arvicanthis* spp. rodents from the Iringa region (20%) and in Natal multimammate mice (*Mastomys natalensis*) from Arusha (18%) and Morogoro (17%) (Table 1), which suggests that these animals are reservoirs of arenaviruses. Titers ranged from 16 to 512 and 16 to 4,096 in *Arvicanthis* spp. rodents and *M. natalensis* mice, respectively. Peak prevalence in *M. natalensis* mice was found on the campus of the Sokoine University in Morogoro (23.7% of 746 animals collected over several seasons).

**Table 1:** Detection of African arenavirus-specific antibodies in small mammals in Tanzania, 1985–1989*

Genus	Antibody detection† by region (no. positive/no. tested)									Total
	**Arusha**	**Iringa**	**Lindi**	Mbeya	**Morogoro**	Mtwara	Ruvuma	Songea	Tanga	
*Acomys*	–	0/3	0/2	0/2	0/57	0/2	–	–	–	0/66
*Aethomys*	–	0/3	0/4	–	0/23	0/11	0/7	0/8	–	0/56
***Arvicanthis***	0/13	**6/30**	–	–	–	–	–	–	0/87	**6/130**
*Cricetomys*	–	–	–	–	0/35	–	–	–	–	0/35
***Lemniscomys***	0/5	**1/2**	**1/2**	–	**1/30**	0/2	0/1	–	–	**3/42**
*Lophuromys*	0/3	0/1	–	–	0/3	–	–	–	0/7	0/14
***Mastomys***	**7/39**	0/17	**1/120**	0/12	**181/1,054**‡	0/81	0/8	0/25	0/82	**189/1,438**
***Mus***	–	0/1	–	0/1	**1/47**	–	–	–	–	**1/49**
*Praomys*	–	0/3	–	0/1	0/1	–	–	–	0/1	0/6
*Rattus*	–	–	0/24	0/1	0/49	0/20	0/3	0/15	0/196	0/308
*Tatera*	0/1	0/1	0/32	–	0/127	0/69	0/11	0/3	–	0/244
*Uranomys*	–	–	–	–	0/11	–	–	–	–	0/11
*Sciuridae*	–	–	0/13	–	0/2	–	0/2	–	0/10	0/27
***Crocidura***	–	–	–	–	**1/14**	–	–	–	–	**1/14**
*Petrodomus*	–	–	0/9	–	–	0/18	–	–	–	0/27
13 other genera	–	0/1	0/2	0/7	0/21	0/20	–	–	0/2	0/53
Total	**7/61**	**7/62**	**2/208**	0/24	**184/1,474**	0/223	0/32	0/51	0/385	**200/2,520**

*Positive samples as well as the respective sampling sites and animals are indicated in **boldface**. †Immunofluorescent antibody (IFA) assay was performed with Lassa virus–infected cells (cut-off titer 16). ‡Fifty IFA assay–positive serum samples were randomly selected and tested by immunoblotting. Presence of African arenavirus–specific antibodies, as defined by reactivity with Lassa virus nucleoprotein and glycoprotein 2, was confirmed in 47 serum specimens.

In 2004, *M. natalensis* mice were trapped in a mosaic of maize fields and fallow grassland at the university campus in the city of Morogoro (6°50′34.9794′′S; 37°38′8.232′′E) to identify the virus. The animal voucher specimens were deposited at the Royal Museum of Central Africa, Tervuren, Belgium. RNA was prepared from 10 μL of rodent serum by using the QIAamp Viral RNA kit (QIAGEN, Valencia, CA, USA), and screening was performed by using a pan–Old World arenavirus reverse transcription–PCR (RT-PCR) specific for the large (L) gene (*9*). One of 96 serum samples was positive (no. 3017/2004) (Table 2), and sequencing of the PCR fragment showed a new arenavirus sequence. The virus was isolated in Vero cells and called Morogoro virus (strain 3017/2004).

**Table 2:** Prevalence of Morogoro virus and Morogoro virus–specific antibodies in *Mastomys natalensis* mice from Morogoro University campus, Tanzania

Specimen and year of sampling	No. samples	No. (%) virus positive (PCR)	No. (%) antibody positive*	No. (%) antibody plus virus positive
Serum 2004	96	1 (1)†	42 (44)	0
Liver 2004	303	12 (4)†	–	–
Serum 2007	63	4 (6)‡	40 (63)	3 (5)§

*By immunofluorescent antibody (IFA) assay, performed with Morogoro virus-infected cells (cut-off 32). †Testing was performed with universal Old World arenavirus large (L) gene reverse transcription–PCR (*9*). ‡Testing was performed with Morogoro virus–specific L gene RT-PCR using primers MoroL3359-forward (5′-AGGATTAGTGAGAGAGAGAGTAATTC-3′) and MoroL3753-reverse (5′-ACATCATTGGGCCCCACTTACTATGGTC-3′). §Titers ranged from 64 to 512.

For sequencing, the isolate was propagated in T75 flasks, virus particles in supernatant were pelleted by ultracentrifugation, and RNA was isolated by using the QIAamp Viral RNA kit (QIAGEN). The entire 3.5-kb small (S) RNA segment was amplified by RT-PCR as described previously (*10*). The 7-kb L RNA segment was amplified in 2 fragments by using a long-range RT-PCR protocol and primers targeting the conserved termini of L RNA and Morogoro virus–specific primers designed on the basis of the sequence of the fragment detected by RT-PCR screening. By using the PCR products as a template, short overlapping fragments were amplified and sequenced with a set of consensus primers for Old World arenaviruses, and S and L RNA sequences were assembled (GenBank accession nos. EU914103 and EU914104). (Sequences reported in this article have been submitted to GenBank and assigned the following accession numbers: full-length S and L RNA sequences of Morogoro virus, EU914103–04; partial L gene sequences of Morogoro virus, EU914107–22; cytochrome B gene of Morogoro virus-positive *Mastomys natalensis*, EU914105–06.)

**Figure 1:**
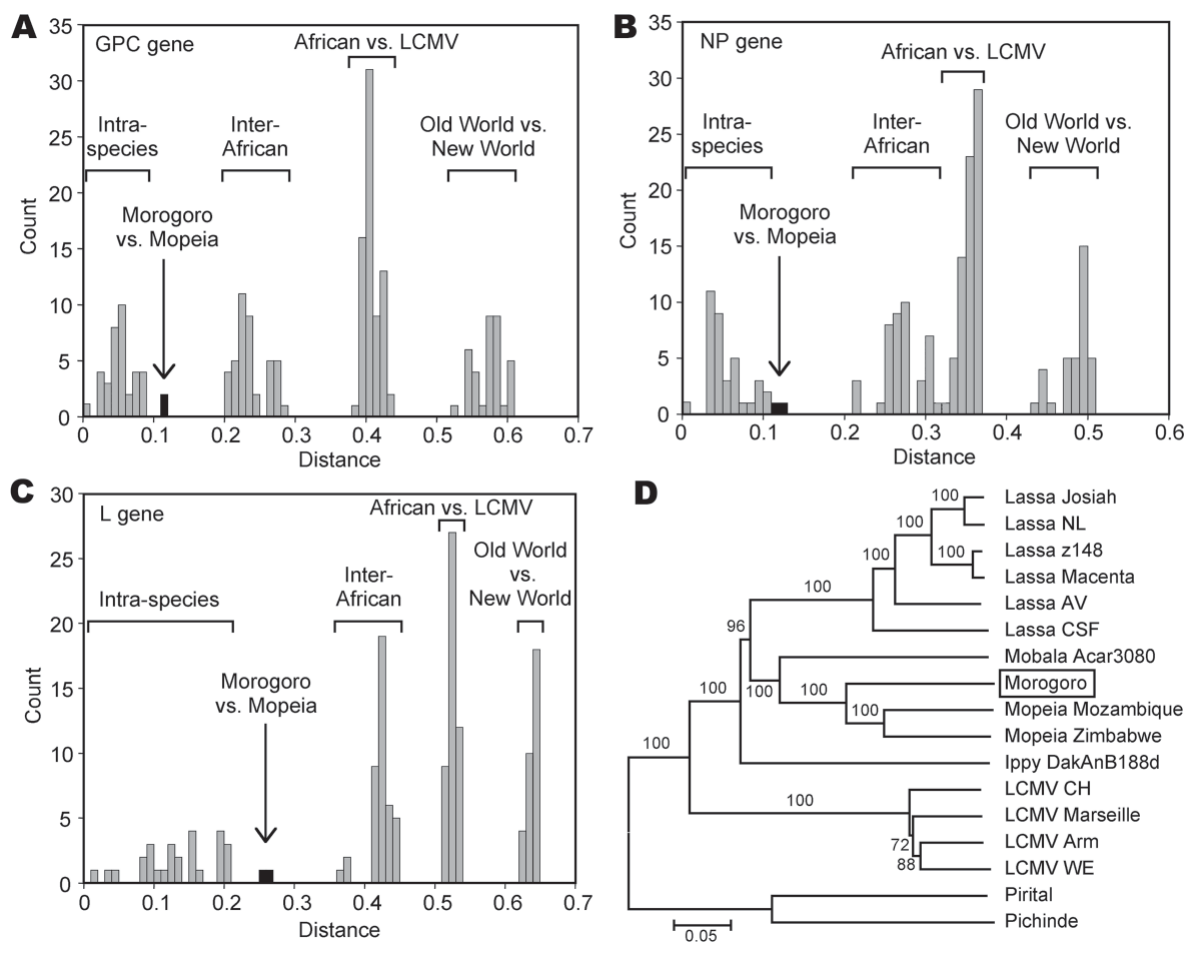
Genetic distances and phylogenetic relationship among arenaviruses, including Morogoro virus. Amino acid sequence diversity was calculated using p distance. Full-length glycoprotein precursor (GPC), nucleoprotein (NP), and large (L) gene amino acid sequences of the following arenaviruses were pairwise compared: Lassa virus (strains Josiah, NL, Z148, Macenta, AV, and CSF), Mobala Acar3080, Morogoro 3017/2004, Mopeia virus (strains Mozambique and Zimbabwe), Ippy DakAnB188d, lymphocytic choriomeningitis virus (LCMV) (strains CH-5692, Marseille, Armstrong, and WE for all genes; Traub and Pasteur for GPC and NP only), Pirital, and Pichinde. Frequency histograms of pairwise distances are shown for A) GPC gene; B) NP gene; and C) L gene. The ranges for intraspecies distances (i.e., pairwise differences between strains of the same virus species); distances between different African arenavirus species; between African arenaviruses and LCMV; and between Old World and New World viruses are marked above the bars. Bars representing the distances between Morogoro virus and the most closely related viruses (Mopeia virus strains) are filled in black. D) Phylogeny of Old World arenaviruses based on full-length L gene amino acid sequences. The tree was inferred by using the neighbor-joining method implemented in the MEGA software package (www.megasoftware.net). The New World arenaviruses Pirital and Pichinde were used as outgroups. Numbers represent bootstrap support (1,000 replications). Identical trees with respect to the phylogenetic position of Morogoro virus (shown in the box) were obtained with full-length GPC and NP amino acid sequences (not shown). Scale bar indicates nucleotide substitutions per site.

Full-length amino acid sequences of glycoprotein precursor (GPC), nucleoprotein (NP), and L protein of Morogoro virus were aligned with published Old World arenavirus sequences and pairwise p distances were calculated. Morogoro virus showed genetic similarity to strains of Mopeia virus that were circulating in Mozambique (*4*) and Zimbabwe (*5*). A close relationship between both viruses was also demonstrated by phylogenetic analysis using GPC, NP, and L gene sequences (Figure 1, panel D, and data not shown). Both viruses are sister taxa, sharing a common ancestor with Mobala virus.

Although the distances between Morogoro and Mopeia virus in the amino acid sequence of GPC (12%), NP (12%–13%), and L gene (26%) were higher than intraspecies differences among known African arenaviruses (i.e., pairwise differences between strains of the same species; <11% in GPC and NP; <21% in L), they did not reach the level of interspecies distances (>20% in GPC and NP; >37% in L) (Figure 1, panels A–C). Therefore, we currently consider Morogoro virus a subspecies of Mopeia virus rather than a new arenavirus species. This classification is supported by the fact that both viruses share the same host. Sequencing of the mitochondrial cytochrome b gene of rodent liver samples positive for Morogoro virus confirmed that its natural host is *M. natalensis* mice (GenBank accession nos. EU914105 and EU914106).

An additional 303 ethanol-preserved liver samples and 63 serum samples were collected in 2004 and 2007, respectively. Liver tissue (≈3 mg) was homogenized by using a bead mill. Cell debris was pelleted by centrifugation, and RNA was isolated from the homogenate with the RNeasy Mini kit (QIAGEN). Testing by L gene RT-PCR (*9*) showed 16 positive liver and serum samples, which indicated a virus prevalence in the *M. natalensis* population of ≈4% (Table 2). PCR fragments were sequenced (GenBank accession nos. EU914107–EU914122), and Morogoro virus was isolated in cell culture from all 4 PCR-positive serum samples obtained in 2007. Morogoro virus–specific antibodies in serum samples from 2004 and 2007 were measured by IFA assay using Vero cells infected with Morogoro virus. The antibody prevalence was ≈50%, which compares quite well with the 23% prevalence determined in this area 20 years before. In some animals, virus and antibodies were detected (Table 2).

**Figure 2:**
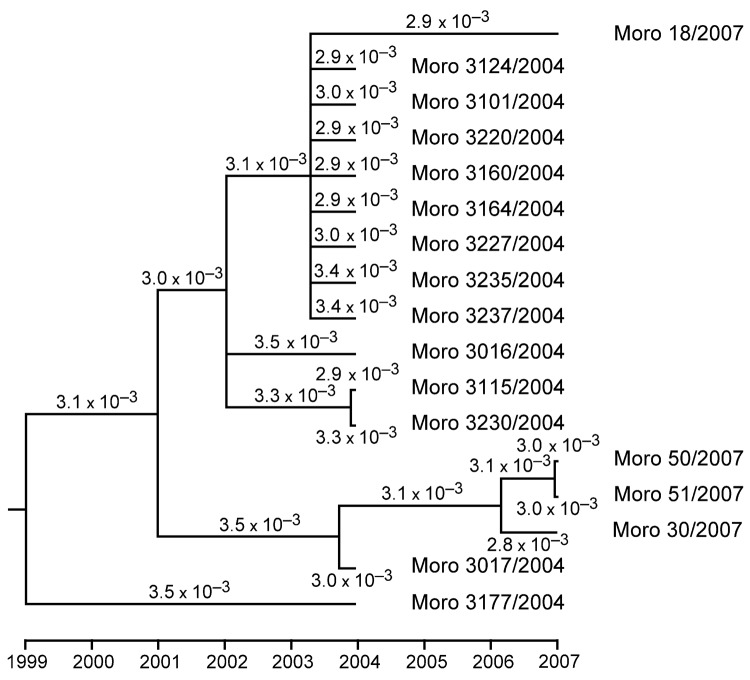
Phylogenetic tree and molecular clock of Morogoro virus based on partial large gene sequences of 17 strains (340 nucleotides; GenBank accession nos. EU914104 and EU914107–EU914122). Phylogeny was inferred with the BEAST v1.4.8 package (*11*) under assumption of a relaxed lognormal molecular clock and general time reversible substitution model with gamma-distributed substitution rate variation among sites. Branches with posterior probability <0.5 were collapsed. The substitution rate per site and year is indicated for each branch. Node ages and rates are median values. Variation in rates among branches is low as calculated with Tracer program (beast.bio.ed.ac.uk/Tracer) indicating a molecular clock in the evolution of Morogoro virus. The same tree topology with similar substitution rates was obtained when assuming the Hasegawa-Kishino-Yano substitution model (not shown).

The availability of Morogoro virus L gene sequences from 2004 and 2007, originating from the same host population (trapping sites <1 km apart), provided us with the opportunity to estimate the molecular clock rate for this virus. Phylogenetic reconstruction was performed with the BEAST version 1.4.8 package (http://beast.bio.ed.ac.uk) (*11*) under the assumption of a relaxed lognormal molecular clock and general time reversible (GTR) or Hasegawa-Kishino-Yano (HKY) substitution model with gamma-distributed substitution rate variation among sites (Figure 2 and data not shown). Analysis was run for 2 million Markov chain Monte Carlo steps, which yielded a reliable set of data as verified with the TRACER program (http://tree.bio.ed.ac.uk/software/tracer). Based on GTR and HYK model, 3.2 × 10^–3^ and 3.4 × 10^–3^ substitutions per site and year (95% interval of highest posterior density 1.1–6.6 × 10^–3^), respectively, were calculated.

## Conclusions

A serologic survey in small mammals from Tanzania identified a hot spot of arenavirus circulation in Morogoro in the late 1980s. This work is being published now because early attempts to substantiate the existence of the virus failed. The identification of the virus was facilitated by a recently developed pan–Old World arenavirus PCR (*9*) that also led to the discovery of new arenaviruses in rodents from West Africa (*7*). Only a small percentage of *M. natalensis* mice carry Morogoro virus, and a large proportion shows specific antibodies, which indicates that most animals clear the virus during life. Viruses and antibodies, which are presumbably directed to nucleocapsid proteins, also co-circulate, as seen in hantavirus infection in rodents (*12*). Detection of Morogoro virus in the liver is consistent with the organ tropism of Lassa virus in *M. natalensis* mice (*13*). In agreement with studies on Lassa virus strains, the largest genetic distance between Morogoro and Mopeia virus was seen in L gene, which contains several highly variable regions (*14*).

The clock rate estimate of 3 × 10^–3^ for Morogoro virus L gene is in agreement with that of other RNA viruses (*15*), although it must be interpreted with caution, given that the difference in date between the samples is not large. The tree topology did not correlate with geographic or ecologic sampling data.

The pathogenicity of Morogoro virus for humans is not known, though its phylogenetic clustering with African arenaviruses that are not linked with human disease (*4*–*6*) and the absence of hemorrhagic fever in the area suggest that it does not cause severe disease. Hospital-based investigations are required to estimate the public health relevance of this virus.
